# Links Between Rumination and Sleep Quality Among Older Adults: An Examination of the Role of Social Support

**DOI:** 10.1093/geroni/igaa057.1853

**Published:** 2020-12-16

**Authors:** Christina Marini, Stephanie Wilson, Suyoung Nah, Lynn Martire, Martin Sliwinski

**Affiliations:** 1 Adelphi University, Garden City, New York, United States; 2 Southern Methodist University, Dallas, Texas, United States; 3 The Pennsylvania State University, State College, Pennsylvania, United States; 4 The Pennsylvania State University, University Park, Pennsylvania, United States; 5 Penn State University, University Park, Pennsylvania, United States

## Abstract

Rumination is a maladaptive coping strategy that gives rise to and sustains stress. Individuals who ruminate more, therefore, tend to sleep more poorly. Studies of rumination and sleep often neglect the role of social context. Social support may buffer the degree to which rumination predicts poorer sleep quality. Further, individuals with more support may ruminate less, resulting in better sleep quality. Finally, rumination may also erode social support, resulting in poorer sleep quality. The current study tested these three hypotheses within a sample of 131 partnered older adults. We examined support from spouses and friends/family separately. Findings indicated that spousal (not family/friend) support buffered the negative association between rumination and sleep quality. Neither type of support predicted rumination; however, rumination predicted lower levels of family/friend (not spousal) support. Thus, spousal support protects older adults’ sleep quality from rumination, and support from their peripheral ties may be more vulnerable to rumination.

